# Empirical Evidence for the Developmental Origins of Autonomic Stress Regulation: An Attachment Account

**DOI:** 10.1002/dev.70195

**Published:** 2026-08-27

**Authors:** Stefania V. Vacaru, Victoria L. Zhu, Greeshma Giridas, Lukas Spiess, Aicha Mazrina, Theodore E. A. Waters

**Affiliations:** ^1^ Department of Psychology New York University Abu Dhabi Abu Dhabi UAE; ^2^ Faculty of Behavioral and Movement Sciences, Clinical Child and Family Studies Vrije Universiteit Amsterdam Amsterdam The Netherlands; ^3^ Institute of Child Development University of Minnesota Minneapolis Minnesota USA; ^4^ Spiess Solution Zutphen The Netherlands; ^5^ Department of Applied Psychology New York University New York New York USA

**Keywords:** attachment security, autonomic nervous system, caregiving quality, heart rate variability, secure base script knowledge, skin conductance level, stress regulation

## Abstract

The quality of the developmental context is proposed to calibrate lifelong stress regulation. Integrating attachment theory with the adaptive calibration model, we tested whether adults exhibit distinct autonomic stress profiles and whether profile membership is associated with attachment security. Adults (*N* = 110; 57% female; *M*_Age_ = 20.46 years) completed the attachment script assessment (ASA) while heart rate variability (HRV) and skin conductance level (SCL) were continuously recorded as indices of parasympathetic and sympathetic activity. Latent profile analysis (LPA) and K‐Means clustering were employed to identify autonomic profiles, and multinomial models were used to test ASA as a predictor of profile membership. Three profiles emerged from the interaction between HRV and SCL activation. In multinomial models with the buffered profile as reference, higher attachment security was associated with lower odds of the unemotional profile across both LPA and *K*‐Means (OR = 0.49 and OR = 0.45), whereas the sensitive and buffered profiles did not differ. An exploratory interaction with sex emerged in the latent profile solution, with females showing higher ASA scores than males within the sensitive profile. Findings support the hypothesis that autonomic stress profiles are linked to attachment security, with lower secure base script knowledge associated with the more dysregulated unemotional profile.

## 1 Introduction

From birth, infants rely on caregivers to maintain homeostasis and recover from stress (Feldman 2007, 2009; Rheinheimer 2025; Rheinheimer et al. 2025). Consistent with attachment theory (Bowlby 1969), the biological systems of infants and caregivers are suggested to operate as co‐regulatory units, with caregivers acting as external regulators of the infant's immature stress system (Brett et al. 2024; Verde‐Cagiao et al. 2022). Over time, these systems become gradually more independent while still reflecting the regulatory imprint of early co‐regulation experiences (Aureli et al. 2022). Through repeated interactions, infants form expectations of their caregiver's support and availability, which in turn guide stress regulation (Ehrlich and Cassidy 2019).

### 1.1 Adaptive Calibration Model and Caregiving Ecology

The adaptive calibration model (ACM; Del Giudice et al. 2011; Ellis and Giudice 2014) provides an explanatory framework for how the early caregiving environment shapes individual differences in stress responsivity. The model proposes that during sensitive developmental periods, repeated experiences of stress and co‐regulation yield responsivity patterns tailored to environmental demands. This conditional adaptation process enables developing organisms to adjust their physiological and behavioral strategies in response to their caregiving ecology (Ellis et al. 2017). Accordingly, early caregiving environments provide the primary context through which an infant's autonomic nervous system calibrates to its expected level of safety, predictability, and social support (Gunnar 2017; Hostinar and Gunnar 2013). The body dynamically orchestrates the coordinated activation of both branches of the autonomic nervous system, the sympathetic and parasympathetic branches, enabling flexible adaptation to both safe and threatening contexts (McCorry 2007).

The ACM specifies four developmental profiles of responsivity that link early caregiving context to profiles of physiological regulation (Del Giudice et al. 2011). In secure, low‐stress contexts, a *sensitive profile* develops, marked by high parasympathetic responsivity that allows efficient regulation of arousal and recovery, coupled with moderate‐to‐high sympathetic activation that supports responsiveness without overactivation. In moderately demanding but predictable environments, a *buffered profile* emerges, characterized by a balanced interplay between moderate sympathetic and parasympathetic activation, maintaining stability under challenge while preventing high physiological cost. Under conditions of chronic threat or unpredictability, a *vigilant profile* is characterized by heightened sympathetic dominance and reduced parasympathetic modulation, resulting in increased stress reactivity and slower physiological recovery. In contrast, in harsh or neglectful contexts, an *unemotional profile* emerges, characterized by blunted responsivity across both autonomic branches, reflecting a hyporeactive configuration that minimizes energetic expenditure and dampens arousal in persistently adverse environments.

The ACM posits sex‐differentiated developmental pathways. Similar environments are thought to elicit distinct calibrations in males and females due to differences in hormonal sensitivity and maturation timing (Del Giudice et al. 2011, 2012). These distinctions are expected to emerge in more dysregulated profiles, that is, individuals with vigilant and unemotional characteristics, as pubertal processes amplify sex‐specific patterns of autonomic responsivity. Accordingly, males have been shown to exhibit relatively higher sympathetic activation, and females show greater parasympathetic regulation.

Empirical support for ACM's hypothesized stress responsivity profiles remains inconclusive. In school‐age children, a four‐profile taxonomy of the sympathetic and parasympathetic branches of the autonomic nervous system emerged, with family (un)predictability predicting profile membership (Del Giudice et al. 2012). In a male adolescent sample, evidence suggested the presence of both three‐ and four‐profile models in relation to familial and ecological environments, such as socioeconomic status (Ellis et al. 2017). By contrast, earlier studies across two independent children samples identified three profiles: sensitive, buffered, and unemotional (Ellis et al. 2005). Evidence for multiple profiles also extends into early development. In 6‐month‐old infants, combined behavioral and physiological measures revealed four distinct profiles, with more regulated profiles linked to lower attachment avoidance at 1 year (Qu and Leerkes 2018). A study with 2‐month‐olds, however, identified three profiles of stress responsivity: sensitive, buffered, and vigilant, with maternal sensitivity predicting higher parasympathetic regulation in the sensitive profile (Girod et al. 2024). Taken together, this evidence suggests that the early emergence of stress regulation profiles is linked to the quality of early caregiving. However, it remains unclear whether these effects extend into adulthood.

### 1.2 Attachment Modulation of Stress Physiology

This body of work collectively suggests that stress regulation is organized around distinct profiles, which are influenced by their caregiving ecology. The ACM emphasizes early caregiving as a key context for calibrating stress systems, aligning with attachment theory's view that early caregiver–infant interactions provide the foundation for lifelong regulatory patterns across socio‐emotional (Vacaru et al. 2019, 2020, 2022; S. Waters et al. 2010) and psychobiological domains (Rheinheimer et al. 2025; Vacaru et al. 2025a, 2025b). Bowlby and Ainsworth (Ainsworth 1978; Bowlby 2003) define attachment relationships as enduring biologically rooted affective bonds between an infant and their primary caregiver. Through innate behaviors such as crying, clinging, and seeking proximity, the infant maintains closeness to an adult attachment figure who provides comfort, safety, and stress regulation.

The primary attachment figure functions as both a secure base from which the child can explore their environment and as a safe haven to return to in times of distress (E. Waters and Cummings 2000; H. Waters and Waters 2006). This secure base relationship provides a balance among exploring the environment, maintaining proximity, and seeking support in times of need (Sroufe and Waters 2007).

More recently, evidence has shown that these internalized expectations of caregiver availability take the form of a cognitive script, namely, the *secure base script* (H. Waters and Waters 2006). This script summarizes a prototypical sequence in which an attachment figure provides support in times of distress, enabling the individual's return to exploration. As reviewed by T. E. A. Waters et al. (2025), the secure base script serves as a heuristic that organizes attachment‐relevant experiences and guides behavior in contexts of support‐seeking and stress regulation across the lifespan. In this view, secure base script knowledge (SBSK) represents a central mechanism through which early caregiving experiences are encoded, generalized, and carried forward into adult physiological and behavioral responses to stress (Groh et al. 2024; Xu and Groh 2023).

The legacy of early caregiving for attachment is suggested to extend throughout the lifespan (e.g., Belsky and Fearon 2002). A core assumption of attachment theory is that attachment expectations formed early in life are relatively stable, serving as an enduring influence that underlies temporary variations in attachment (Fraley et al. 2011; Jones et al. 2018; T. E. A. Waters et al. 2022). Meta‐analytically, the longitudinal stability of attachment security has demonstrated a robust yet modest effect (*r* = 0.39; Fraley 2002). Evidence from a prospective longitudinal study revealed a significant association between attachment security assessed in the first 3 years of life and later attachment security in adulthood (Groh et al. 2014; Steele et al. 2014), and a 20‐year longitudinal study found that 72% of infants' attachment classifications at 12 months of age persisted into adulthood (E. Waters et al. 2000). Likewise, observed caregiver sensitivity in childhood predicts SBSK in adulthood (e.g., Steele et al. 2014; T. E. Waters et al. 2017). SBSK stability was established from middle childhood to adolescence (T. E. A. Waters et al. 2019) and from early adolescence to midlife (T. E. A. Waters et al. 2021).

Extensive empirical evidence has documented the association between quality of attachment relationships and stress reactivity biomarkers in infancy (Groh and Narayan 2019; Zvara et al. 2025), throughout adolescence (e.g., Beijersbergen et al. 2008), extending into adulthood (Gallistl et al. 2025; Roisman et al. 2004; Vacaru et al. 2025a), showing that more secure attachment translates to better physiological regulation, in terms of arousal, reactivity, and recovery. Yet most of this evidence assesses attachment and physiology within the same developmental period, thereby characterizing their concurrent association. The few studies that have introduced a temporal lag assessed mostly the neuroendocrine domain. Infant attachment security has been associated with cortisol reactivity at preschool age (Müller et al. 2022) and in adolescence (Fearon et al. 2017). One study has extended this prospective approach to the autonomic nervous system, in which infant attachment predicted attenuated parasympathetic reactivity in middle childhood in a high‐risk sample (Tabachnick et al. 2021). The prospective link between early attachment and autonomic regulation in adulthood thus remains largely uncharted.

Whether early attachment leaves a lasting imprint on physiology may also depend on the continuity of the autonomic system. Resting parasympathetic tone shows moderate rank‐order stability across multi‐year intervals, from the preschool years (Calkins and Keane 2004) through middle childhood (Hinnant et al. 2011) and into adolescence (El‐Sheikh 2005), and individual differences in resting cardiac activity behave in a trait‐like manner across the retest intervals examined in adulthood (Seidman et al. 2024). This stability is found mainly at the resting level. Reactivity, the dynamic withdrawal and recovery that the present profiles index, is considerably less stable, with little continuity from infancy into early childhood (Bornstein and Suess 2000; Alkon et al. 2011) and lacking stability across the toddler‐to‐adolescent span (Dollar et al. 2020). Electrodermal individual differences are likewise described as trait‐like in adulthood (Crider 2008), though the developmental evidence is sparser. Furthermore, most studies rely on bivariate associations with single indices of reactivity, leaving two gaps: (i) evidence of the ACM's four proposed profiles remains scarce, and (ii) the role of SBSK as an attachment mechanism within ACM has not been examined.

### 1.3 Current Study

Guided by the ACM and attachment theory, we examine whether attachment security is linked to distinct autonomic profiles of stress reactivity that map onto the sensitive, buffered, vigilant, and unemotional profiles. Our work extends beyond the use of single biomarkers to identify stress profiles resulting from the interplay between sympathetic and parasympathetic activity in response to a stressor (*Aim 1*). We hypothesize that profiles of stress regulation will vary systematically with adults’ attachment security, operationalized as SBSK (H. Waters and Waters 2006; T. E. Waters and Roisman 2019) (*Aim 2*). Specifically, lower SBSK scores, reflecting attachment insecurity, will be associated with profiles of heightened stress dysregulation (i.e., unemotional, vigilant). Higher SBSK scores, reflecting attachment security, are expected to predict more regulated profiles (i.e., sensitive, buffered). Finally, as an exploratory aim, we test whether biological sex moderates the relation between SBSK and profile membership (*Aim 3*). Based on evolutionary and physiological accounts of sex‐differentiated stress regulation, we expect that within dysregulated profiles (i.e., vigilant, unemotional), males will exhibit greater sympathetic dominance, whereas females will show greater parasympathetic regulation.

## 2 Materials and Methods

### 2.1 Participants

One hundred and ten adults (*M*_Age_ = 20.46, SD_Age_ = 1.67; females = 57.3%, where SD is standard deviation) were recruited through New York University Abu Dhabi's (United Arab Emirates) participant recruitment SONA system. The study was approved by the NYUAD Institutional Review Board (HRPP‐2023‐46) and was performed in accordance with the World Medical Association Declaration of Helsinki. An a priori power analysis conducted using G*Power 3.1 (*α* = 0.05, power = 0.90, small‐to‐medium effect size *f* ^2^ = 0.15) indicated that a sample of 99 participants would be required to detect significant effects in a multiple regression model with three predictors. We tested 10% above the required number to mitigate potential data loss. Because our primary analyses used latent profile analysis (LPA) and a subsequent multinomial logistic regression, for which no power calculation is available, we treated this only as a planning benchmark and conducted a Monte Carlo simulation in R with 1500 datasets using the multinom function (nnet package). Following Hoenig and Heisey (2001), this approach estimates the smallest effect a design can reliably detect. Models reached roughly 80% power only for large associations, corresponding to odds ratios of about 0.29 per SD for the sensitive‐versus‐buffered contrast, 0.32 for the unemotional‐versus‐buffered contrast, and 0.19 for the sensitive‐versus‐unemotional contrast. Prior to participating in the study, participants provided written informed consent, and the privacy rights of human subjects were observed. Descriptive statistics and sample characteristics are reported in Table 1.

**TABLE 1:** Demographic characteristics of the sample (*N* = 110).

	%	*M*	SD	Range
Age		20.46	1.67	18–26
Sex	40.9% male 57.3% female 1.8% nonbinary			
Ethnicity	41.8% South/Central Asian 16.4% East/Southeast Asian 13.6% White Caucasian 11.8% Middle Eastern/North African 8.2% Black African American 5.5% Hispanic/Latino 2.7% Mixed			
Education level	29.1% first year undergrad 13.6% second year undergrad 23.6% third year undergrad 28.2% fourth year undergrad 5.5% postgraduate			
Medical condition	83.6% none 12.7% yes			
Body mass index		22.61	4.02	15.06–36.20
Smoking	86.4% no 12.7% yes	0.47	2.17	0–20
Alcohol	65.5% no 30.9% yes	0.48	1.46	0–10
SBSK		3.66	1.06	1.63–6.00

Abbreviations: *M*, mean; SBSK, secure base script knowledge; SD = standard deviation.

### 2.2 Data Acquisition and Procedure

Participants completed a 2‐h laboratory session. Prior to the start of the session, they were instructed to avoid strenuous exercise and caffeine for 3 h. Electrode application was performed either by a female researcher or by participants themselves (*n* = 13) in a private room with mirrors and visual guides, based on their preference. Physiological data, including electrocardiogram (ECG) and electrodermal activity (EDA), were recorded at a sampling rate of 500 Hz using a Mindware Mobile Impedance Cardiograph (Mindware Technologies, Westerville, OH) and BioLab 3.4.1 acquisition software. For the ECG, electrodes were placed in a standard Lead‐II configuration after the skin was cleaned with alcohol wipes. For EDA, participants washed their hands without soap, and electrodes were placed on the palmar surface of the nondominant hand. Following a 5‐min baseline, participants completed the attachment script assessment (ASA). Event markers, manually inserted by the experimenter in Biolab, indicated the start and end of the baseline and each ASA story. Demographic questionnaires were administered via Qualtrics. Participants received course credit (2 credits) or monetary compensation (100 AED/25 USD) for their time.

### 2.3 Instruments

The adolescent ASA (H. Waters and Waters 2006) served as both a measure of SBSK and the stress‐induction task, aligning with recent work on bivariate associations between the ASA and physiological stress (Groh et al. 2024). The ASA consisted of five prompt‐word outlines: one neutral scenario (*Afternoon Shopping*) and four attachment‐related scenarios (*Acne* [females], *Bad Haircut* [males], *Not Invited to a Party*, *Studying for an Exam*, and *Soccer Game*). Participants were instructed to tell the best possible story they could, based on each outline, using a first‐person perspective. All narratives were audio‐recorded and transcribed verbatim.

An expert coder trained four coders on a subset of ASA stories (approximately 20 per story type) until they achieved high interrater reliability (ICCs = 0.79–0.95). Once reliability was achieved, participants’ ASA stories were divided evenly between two pairs of coders, with each ASA story being double‐coded. All discrepancies exceeding 1 point were discussed and resolved by consensus. Stories were rated on a 7‐point scale indexing the degree of SBSK in the narratives (Dykas et al. 2006). The total SBSK score is computed as the mean score of the four attachment stories. Scores of 4–7 reflected stories that incorporated SBSK elements, with higher scores indicating more elaborated secure base descriptions. Scores of 3 denoted event‐focused stories with minimal SBSK content, while scores of 1–2 were given to atypical or disjointed narratives that deviated from the secure base script. Interrater reliabilities across all stories were good to excellent (ICCs = 0.77–0.92), and internal consistency across the attachment‐related stories was acceptable (Cronbach's *α* = 0.72).

*Covariates*: Participants were asked four health‐related questions: (1) height and weight from which BMI was calculated [weight (kg)/[height (m)]^2^], (2) Do you have a health condition? (yes, no, prefer not to say), (3) Do you smoke? (yes, no, prefer not to say), and (4) Do you drink alcohol? (yes, no, prefer not to say). Additionally, participants were asked how long ago they had consumed caffeine and exercised, each operationalized as 1 = < 12 h before participation and 2 = > 12 h before participation.

### 2.4 Data Preprocessing

Signal preprocessing for both ECG and EDA was performed in Python (version 3.10.14) using the NeuroKit2 Python Toolbox for Neurophysiological Signal Processing (version 0.2.10; Makowski et al. 2021). Due to technical problems, the EDA data from nine participants were not recorded. Data from one additional participant could not be analyzed due to missing markers.

*ECG*: First, the entire ECG time series was cleaned to remove noise and improve the accuracy of subsequent peak detection, using NeuroKit's *ecg_clean* function. This applies a fifth‐order 0.5 Hz high‐pass Butterworth filter, followed by a 50 Hz powerline filter. The second preprocessing step involved detecting R‐peaks using NeuroKit's *ecg_peaks* function, which detects QRS complexes based on the steepness of the signal's absolute gradient. R‐peaks are then detected as local maxima in the QRS complexes. Potential artifacts were identified and fixed using an in‐built implementation of the method by Lipponen and Tarvainen (2019). In a third step, the R‐peaks time series was segmented using the event markers for the on and offset of the baseline period and each of the five stories. Finally, peak detection performance was verified by visually inspecting the R‐peaks superimposed on the cleaned ECG signal for all segments of all participants.

*EDA*: The electrodermal signal was cleaned using the NeuroKit's *eda_process* function with “neurokit” as the default method. In the first step, the EDA signal was cleaned using a fourth‐order 3 Hz low‐pass Butterworth filter. In the second step, the cleaned EDA signal was decomposed into tonic and phasic components using second‐order Butterworth filters with a 0.05 Hz cutoff: a low‐pass filter for the tonic component and a high‐pass filter for the phasic component. Afterward, the time‐series signal of the tonic phase was segmented using event markers for the onset and offset of the baseline period and for each of the five stories.

### 2.5 Feature Extraction

Heart rate variability (HRV) was operationalized as the SD of the R‐R intervals, commonly referred to as SDNN (SD of normal‐to‐normal intervals). To account for variability in story durations, it was decided to split each segment, including the baseline, into nonoverlapping 30‐s time windows. If a time window was shorter than 30 s (e.g., due to a story offset), it was retained only if it contained at least 20 R‐peaks. HRV was then calculated separately for each time window within a segment. In the next step, time windows with HRV values more than 2.56 SDs from the participant's mean HRV across all time windows were removed. The HRV values for the remaining time windows were averaged to obtain a single HRV value for the baseline and each of the five stories. To account for individual differences, baseline correction was applied by subtracting the baseline HRV from each story's HRV. Baseline‐corrected HRV values represent the average 30‐s R‐R interval SD (in ms) per story segment, with positive values indicating increased HRV relative to baseline. In contrast, negative values indicate a decreased HRV relative to the baseline.

*Skin conductance level (SCL)*: The average SCL (expressed in µSiemens), also known as tonic SCL, was calculated by averaging the tonic SCL across segments. As before, baseline correction was performed by subtracting the average SCL of the baseline from the average SCL of each story segment. Higher values, therefore, indicate a higher SCL during a story segment than during the baseline period.

### 2.6 Statistical Analysis

Statistical analysis was performed using R (version 4.4.3) and SPSS (version 29). The physiological data were inspected for outliers. For HRV, we identified two extreme values based on a *z*‐score threshold of > 3, which represent physiologically implausible measurements. These were winsorized by replacing them with the second most extreme non‐outlier value, thereby reducing undue statistical influence while preserving relative data rankings. SCL and HRV values were *z*‐scored for cross‐individual comparison. Missing data were handled using the *missForest* function in R (Stekhoven and Bühlmann 2012), which imputes missing values through a nonparametric random forest approach. First, descriptive and correlation analyses were performed.

To test Aim 1, namely, to derive autonomic stress profiles, we employed a finite Gaussian mixture modeling with LPA (Oberski 2016) and *k*‐means clustering (Zakharov 2016). Using both methods, LPA as a model‐based probabilistic approach and *k*‐means as a distribution‐free, variance‐partitioning approach, helped ensure that the profiles were not artifacts of the statistical approach, thereby increasing confidence in the clustering robustness. LPA allowed theory‐driven model selection using fit indices (Akaike information criterion [AIC], Bayesian information criterion [BIC]), likelihood ratio tests (LRT), entropy, and class size criteria, with participants assigned to the profile with the highest posterior probability. In contrast, *k*‐means grouped individuals by minimizing within‐cluster variance and determined the optimal number of clusters by identifying the elbow of the within‐cluster sum of squares (WSS). In this study, the best‐fitting LPA solutions minimized AIC, BIC, and LRT, achieved an acceptable entropy, and retained profiles that accounted for> 5% of the sample. *K*‐means solutions (*k* = 1–6) were evaluated with the elbow method, and convergence was assessed by cross‐tabulation, Sankey visualization, and the adjusted Rand index (ARI).

For Aim 2, separate models were estimated for the LPA‐ and *k*‐means–derived classifications, with profile membership as the dependent variable and SBSK, sex, and their interaction (SBSK × sex) as predictors. To account for small and uneven subgroup sizes, bootstrapped standard errors and bias‐corrected 95% confidence intervals (BCa) were computed from 2000 resamples, yielding robust estimates of model parameters.

For Aim 3, we included the interaction term between SBSK and sex in the model. Prior to computing the interaction term, SBSK scores were *z*‐scored. Potential covariates (age, medical condition, BMI, smoking status, and alcohol use) were also included. Model comparisons used LRTs, followed by planned post‐hoc analyses to unpack significant effects. Significance was set at *α* = 0.05. Two participants identified as nonbinary. While they were retained in the profile analyses, they were excluded from the moderation analyses, since a third category of this size could not be modeled.

## 3 Results

### 3.1 Preliminary Analysis

Descriptive analyses of the four attachment stories and baseline‐corrected physiological indices are presented in Table 2. Pearson and Spearman correlation coefficients among continuous and categorical variables, respectively, are displayed in Table 3.

**TABLE 2:** Descriptive statistics of the SBSK and physiological indices.

	SBSK		HRV		SCL	
	***M***	**SD**	***M***	**SD**	***M***	**SD**
AC/BH	3.64	1.67	−5.071	20.424	5.058	4.269
NITP	3.95	1.47	−4.196	18.631	5.045	4.298
SG	3.47	1.26	−3.483	18.307	5.214	4.343
SFT	3.59	1.37	−5.394	19.517	5.064	4.337
SBSK	3.66	1.06	−4.678	17.905	5.091	4.187

Abbreviations: AC, acne (1–7); BH, bad haircut (1–7); HRV, heart rate variability; NITP, not invited to a party (1–7); SBSK, secure base script knowledge (1–7); SCL, skin conductance level; SFT, studying for an exam (1–7); SG, soccer game (1–7).

**TABLE 3:** Bivariate correlation table.

	1.	2.	3.	4.	5.	6.	7.	8.	9.	10.	11.
1. SBSK	—	0.194*	0.008	0.051	0.069	−0.010	0.008	−0.104	−0.008	0.012	0.009
2. zHRV		—	0.130	0.181	−0.034	−0.051	0.084	−0.033	−0.049	0.062	0.046
3. zSCL			—	−0.006	0.032	−0.147	−0.202*	−0.226*	−0.042	0.596	0.597
4. Age				—	0.089	0.058	0.139	0.367*	0.386**	−0.067	−0.090
5. Sex					—	0.221*	−0.089	0.044	0.130	−0.088	0.069
6. Medical condition						—	−0.053	0.091	0.189	−0.112	0.077
7. BMI							—	0.089	−0.106	−0.052	−0.057
8. Smoking								—	0.359**	−0.139	0.027
9. Alcohol									—	−0.103	−0.043
10. Caffeine										—	0.066
11. Exercise											—

*Note:* Spearman coefficients are reported for categorical variables and Pearson coefficients for continuous variables; sex was coded as 1 = male, 2 = female; medical condition, smoking, and alcohol were coded as not present = 1, present = 2. Caffeine and exercise were coded as 1 = < 12 h before participation and 2 = > 12 h before participation.

Abbreviations: BMI, body mass index; SBSK, secure base script knowledge; zHRV, standardized heart rate variability (winsorized); zSCL, standardized skin conductance level.

*p < 0.05; **p < 0.01.

### 3.2 Profiles of Stress Regulation

To examine the presence of profiles specified by the ACM (Aim 1), we employed two clustering approaches: LPA and *K*‐means clustering.

With the LPA, we tested one to five profiles to identify the best‐fitting model (see Table 4 for fit indices). The AIC values supported a three‐profile solution, whereas the BIC values were identical for both the two‐profile and three‐profile solutions. Because the BIC was identical for the two‐ and three‐profile solutions, the three‐profile solution was retained based on its slightly higher entropy (0.84 vs. 0.82), better theoretical interpretability, and acceptable class sizes (all > 5%).

**TABLE 4:** Fit indices for latent profile analysis models.

Profiles	AIC	BIC	Entropy	Prob min	Prob max	*N* min	*N* max	LRT (*p*)
2	620	**639**	0.82	0.82	0.97	0.15	0.85	−303 (0.02)
3	**612**	639	**0.84**	0.79	0.97	0.09	0.79	**−296 (0.02)**
4	612	647	0.74	0.67	0.93	0.09	0.59	−293 (0.24)
5	617	660	0.62	0.59	0.95	0.09	0.35	−292 (0.70)

*Note:* Best‐fitting values are in bold.

Abbreviations: AIC, Akaike information criterion; BIC, Bayesian information criterion; LRT, likelihood ratio test; *N* min/max, minimum and maximum proportion of participants classified in smallest and largest classes; Prob min/max, minimum and maximum posterior probabilities;

Descriptive statistics of the physiological (HRV, SCL) and behavioral (SBSK) variables for each latent profile are presented in Table 5. Profile 1 (unemotional), which included 11.82% (*n* = 13) of participants, was characterized by the lowest HRV and low‐to‐medium SCL, indicating reduced arousal and autonomic regulatory activity during stress. Profile 2 (buffered) included 79.09% (*n* = 87) of participants, and showed the highest HRV and medium SCL, reflecting high regulatory activity and moderate arousal to the stressor. Profile 3 (sensitive) included 9.09% (*n* = 10) of participants and exhibited medium‐to‐high HRV and the highest SCL, indicating high reactivity to the stressor and moderate‐to‐high regulation. The buffered profile (*n* = 87) contained 35 males, 51 females, and one nonbinary; the unemotional profile (*n* = 13) contained five males and eight females; and the sensitive profile (*n* = 10) contained five males, four females, and one nonbinary.

**TABLE 5:** Descriptive statistics of physiological (HRV, SCL) and behavioral (SBSK) measures across latent profiles (LPA) and *K*‐means classes.

	HRV *M* (SD)	SCL *M* (SD)	SBSK *M* (SD)
Profile 1 (*n* = 13): unemotional	−1.99 (0.62)	0.09 (0.65)	3.03 (1.01)
Profile 2 (*n* = 87): buffered	0.21 (0.69)	−0.25 (0.76)	3.72 (1.03)
Profile 3 (*n* = 10): sensitive	0.74 (0.48)	2.13 (0.46)	3.98 (1.16)
Class 1 (*n* = 21): unemotional	−1.58 (0.74)	0.01 (0.60)	3.13 (0.87)
Class 2 (*n* = 45): buffered	0.22 (0.60)	−0.82 (0.56)	3.95 (1.02)
Class 3 (*n* = 44): sensitive	0.53 (0.60)	0.83 (0.79)	3.63 (1.11)

*Note:* Means and standard deviations of heart rate variability (HRV), skin conductance level (SCL), and secure base knowledge scripts (SBKS) scores are presented separately for latent profiles identified through latent profile analysis (Profile 1 = unemotional, Profile 2 = buffered, Profile 3 = sensitive) and for classes identified through *k*‐means clustering.

**FIGURE 1:**
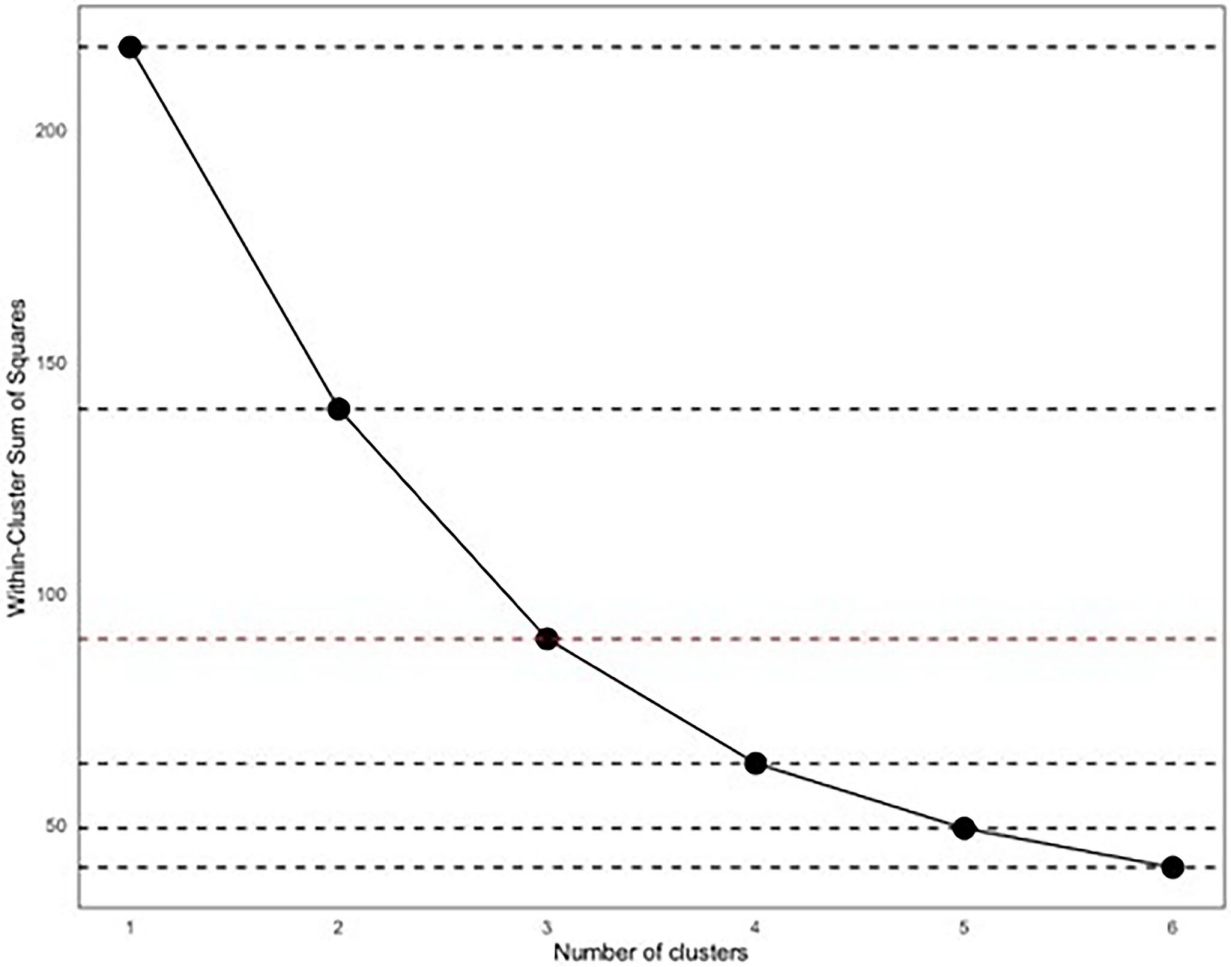
Elbow plot of within‐cluster sum of squares for *k*‐means clustering solutions. The elbow method displays the within‐cluster sum of squares (WSS) for solutions with one to six clusters. The inflection point indicates a drop in the rate of decrease in WSS from three to four clusters, supporting a three‐cluster solution. The horizontal lines represent the change in WSS across successive cluster solutions.

**FIGURE 2:**
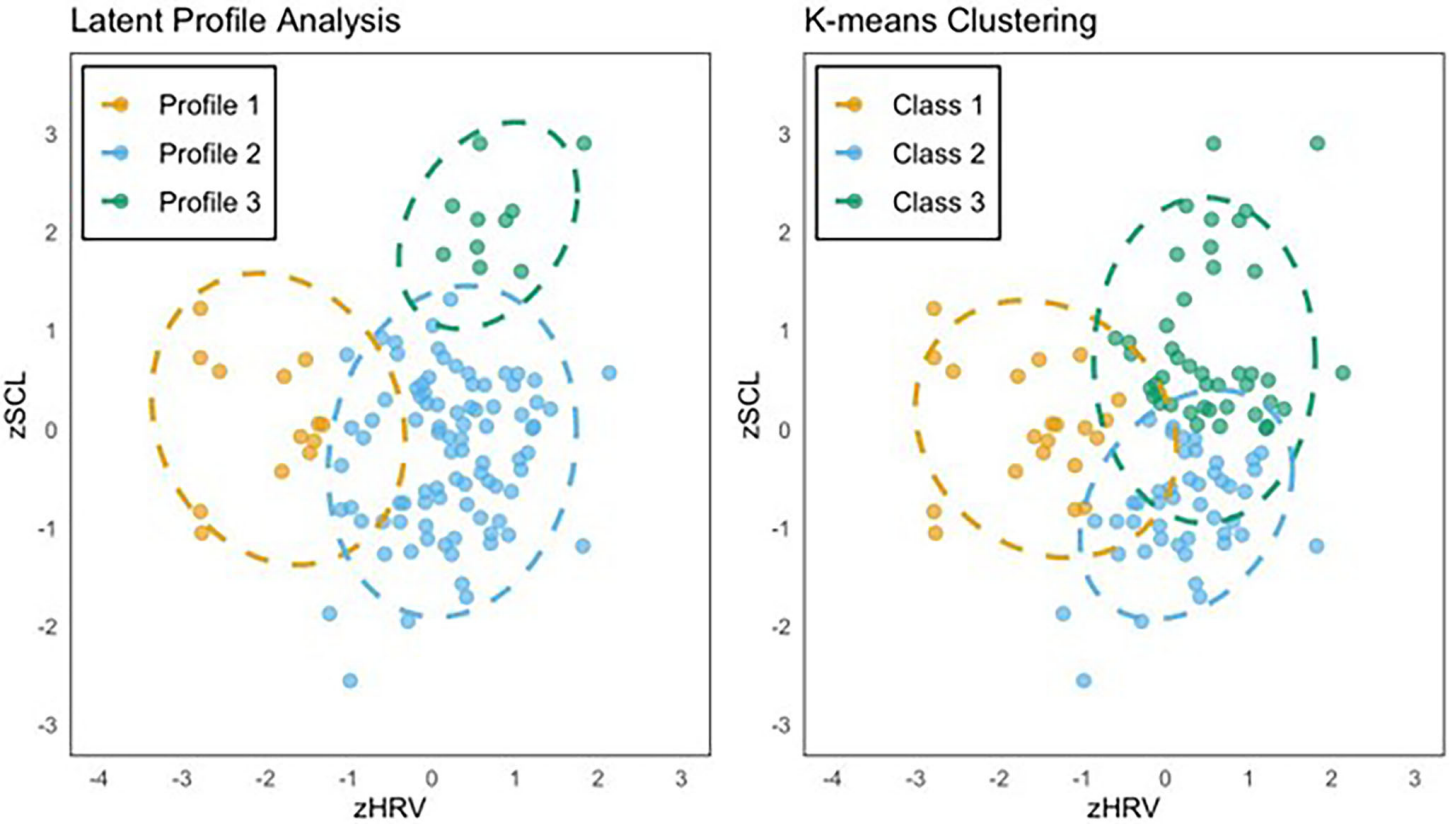
Patterns of HRV and SCL for the latent profile analysis and *K*‐means clustering. Scatterplots show standardized heart rate variability (HRV) and skin conductance level (SCL) scores, colored by latent profiles (left) and classes (right). The left figure presents profiles identified via latent profile analysis (LPA), and the right figure shows classes identified using *K*‐means clustering. Each ellipse depicts the estimated 95% confidence region of the bivariate distribution for that profile/cluster.

Next, to test the robustness of the profile assignment, we conducted a *k*‐means analysis. The WSS was computed for solutions with one to six clusters to determine the optimal number of clusters. The WSS decreased sharply between one and two clusters (from 218 to 139.82) and again between two and three clusters (from 139.82 to 90.24). Beyond three clusters, the reduction in WSS leveled off (from 90.24 to 63.37 for three to four clusters), indicating diminishing returns. Following the elbow method, the inflection point in the three‐cluster solution was taken as the optimal choice (Figure 1). Thus, the three‐cluster solution was selected for subsequent analyses. The profiles are visualized in Figure 2.

**FIGURE 3:**
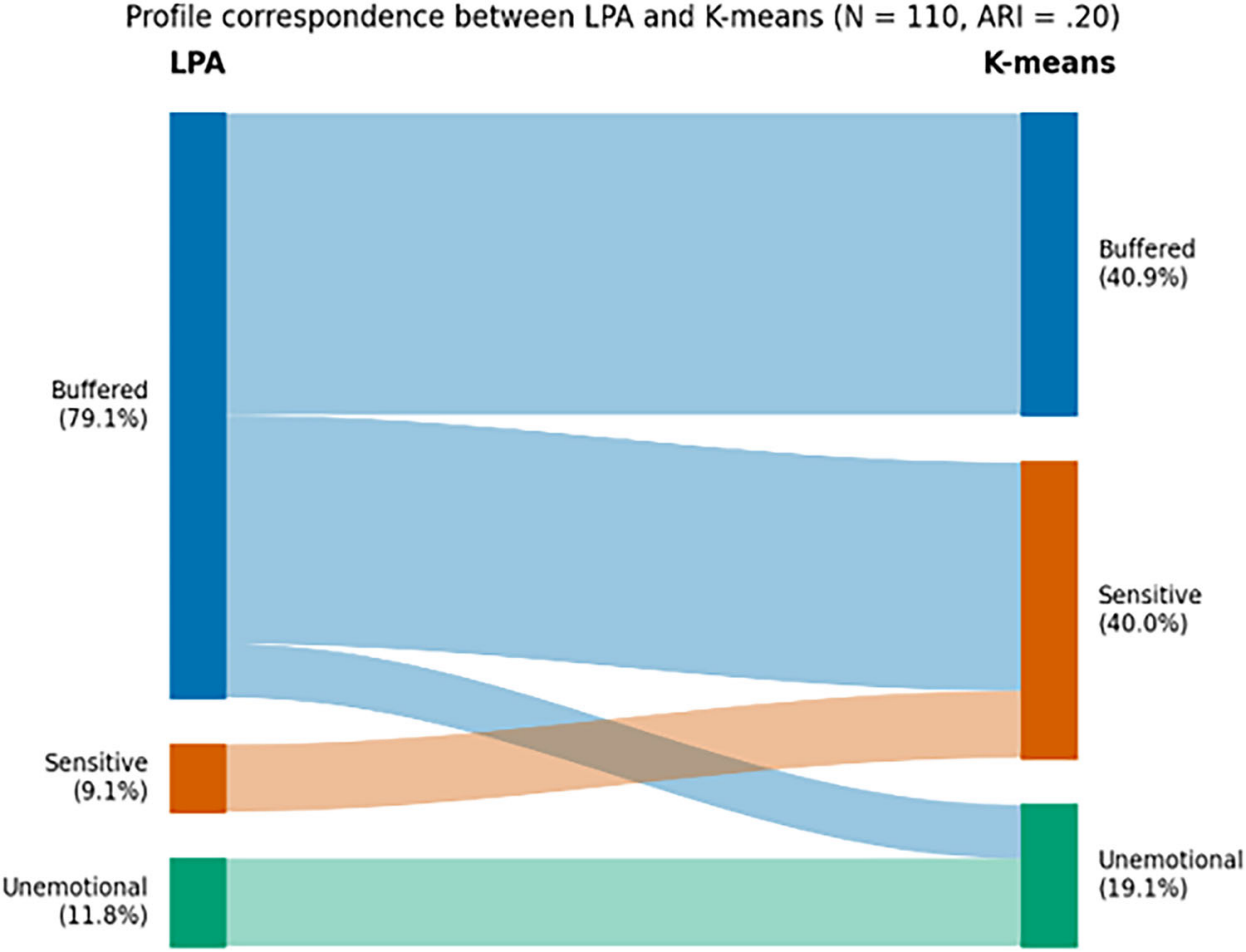
Convergence of LPA and *k*‐means classification results. A Sankey diagram illustrates the overlap between the LPA results and *k*‐means clustering results (rectangles denote profiles in the two analytical classifications; colored banners denote flows from one profile to another across the two methods; the widths of the banners are proportional to the number of overlapping individuals’ profiles in each segment).

To examine the degree of agreement between the latent profile and *k*‐means clustering solutions, participant assignments were cross‐tabulated and visualized in a Sankey diagram (Figure 3). The overlap was quantified using the ARI (= 0.20), indicating overlap above random. The 34 reassigned participants were separated from those who remained buffered almost entirely on sympathetic arousal. Their skin conductance was higher, *M* = 0.45 versus −0.82, *t*(71.5) = 12.90, *p* < 0.001, while their parasympathetic activity did not differ, *M* = 0.46 versus 0.22, *t*(69.9) = 1.73, *p* = 0.088, placing them at an intermediate point on the sympathetic gradient between the buffered and sensitive groups. SBSK did not track this shift. The reassigned participants did not differ from those who remained buffered, *M* = 3.53 versus 3.95, *t*(68.8) = −1.77, *p* = 0.08, or from the sensitive group, *M* = 3.53 versus 3.98, *t*(13.9) = −1.11, *p* = 0.29, and the buffered and sensitive groups did not differ in SBSK overall, *t*(10.7) = −0.68, *p* = 0.51.

### 3.3 SBSK Predicting Stress Reactivity Profiles

To test Aim 2, we performed a multinomial logistic regression examining whether SBSK scores predicted profile membership as determined by the LPA and *K*‐means. Based on the prior analysis, we specified the buffered profile (Profile 2) as the reference category in the multinomial logistic regression because it represented the largest group, providing a stable and theoretically meaningful comparison for evaluating predictors of membership in the smaller unemotional and sensitive profiles. Covariates (age, BMI, medical condition, smoking, and alcohol use) were first examined against a null model, yielding nonsignificant results (all *p*s > 0.125); thus, they were not retained in subsequent models. Second, the model, which included SBSK scores and sex, and their interaction, significantly improved the model fit for the LPA profiles (LRT: *χ* ^2^(6) = 13.57, *p* = 0.035). Results showed a significant main effect of SBSK on group membership (*χ* ^2^(2) = 6.75, *p* = 0.033) and a main effect of sex (*χ* ^2^(2) = 7.37, *p* = 0.025). Relative to the buffered group, lower SBSK scores predicted a higher likelihood of belonging to the unemotional profile, *B* = −0.71, SE = 0.37, *p* = 0.025, Exp(*B*) = 0.49, 95% BCa CI [0.19, 1.08], whereas the sensitive profile did not differ from buffered, *B* = 0.38, SE = 0.43, *p* = 0.386, Exp(*B*) = 1.46, 95% BCa CI [0.50, 2.67].

Results from the *K*‐means additive model showed that SBSK scores improved the model fit for the *k*‐means clusters (LRT: *χ* ^2^(4) = 9.24, *p* = 0.055). The main effect of SBSK was significant *χ* ^2^(2) = 9.01, *p* = 0.011), while the main effect of sex was not *χ* ^2^(2) = 0.30, *p* = 0.862). Relative to the buffered class, lower SBSK scores were associated with lower odds of belonging to the unemotional cluster, *B* = −0.81, SE = 0.29, *p* = 0.005, Exp(*B*) = 0.45, 95% BCa CI [0.24, 0.78] and with lower odds of the sensitive cluster, *B* = −0.30, SE = 0.21, *p* = 0.145, Exp(*B*) = 0.74, 95% BCa CI [0.44, 1.15].

### 3.4 Exploratory Analyses on the Moderation Effect of Sex

For Aim 3, we included the SBSK × sex interaction term in the models with LPA and *K*‐means clustering as dependent variables, separately. The interaction reached the conventional threshold for the LPA solution, *χ* ^2^(2) = 6.83, *p* = 0.033, but not for the *k*‐means solution, *χ* ^2^(2) = 1.35, *p* = 0.51. Because this effect emerged in only one of the two clustering solutions and several profiles contained fewer than 10 participants, we treat it as exploratory and hypothesis‐generating rather than as evidence for a robust sex‐specific effect.

**FIGURE 4:**
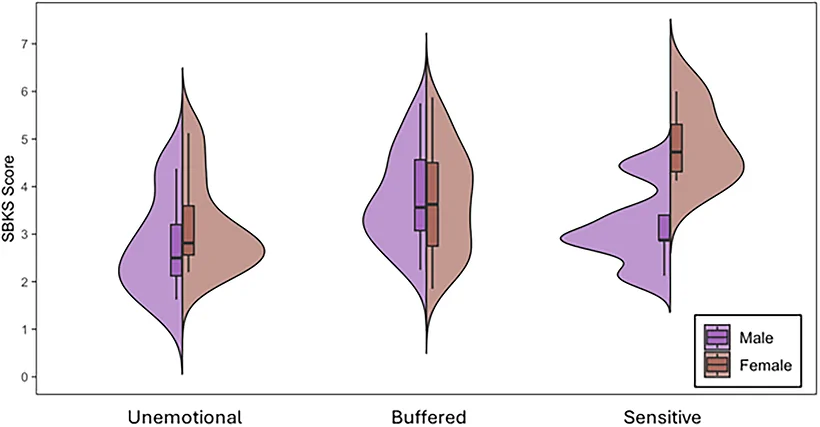
Distribution of SBSK scores across latent profiles split by sex. Violin plots illustrate the distribution and density of scores within each profile, with females shown in brown and males in purple. Median and interquartile ranges are overlaid to highlight central tendency and variability.

Exploratory, follow‐up simple effects tests (univariate contrasts in GLM) for the LPA‐derived profiles showed that boys and girls did not differ in SBSK scores within the buffered profile, *F*(1, 102) = 0.03, *p* = 0.869, *M* difference = 0.04, SE = 0.23, 95% CI [−0.41, 0.48], or the unemotional profile, *F*(1, 102) = 0.54, *p* = 0.466, *M* difference = −0.43, SE = 0.59, 95% CI [−1.59, 0.72]. However, within the sensitive profile, boys scored significantly lower than girls, *F*(1, 102) = 6.36, *p* = 0.013, *M* difference = −1.75, SE = 0.69, 95% CI [−3.13, −0.37]. For an illustration of the moderation analysis, see Figure 4.

## 4 Discussion

We examined autonomic profiles of stress responsivity, as indexed by HRV and SCL, during an attachment‐based narrative task designed to elicit internalized scripts of early attachment representations, and their association with SBSK. Our findings partially support the ACM model (Del Giudice et al. 2012), providing more evidence for the three‐profile over the four‐profile solution, and replicating previous findings with children (Ellis et al. 2005) and infants (Girod et al. 2024). The robustness of the findings was established using LPA and *k*‐means clustering methods. We hypothesized that lower SBSK, reflecting attachment insecurity, would be associated with the more dysregulated autonomic profiles, and that higher SBSK, reflecting attachment security, would be associated with the more regulated profiles. The results partly confirmed this. Lower SBSK was associated with higher odds of the unemotional profile relative to the buffered profile, consistently across both the latent profile and *k*‐means solutions, whereas the two regulated profiles, buffered and sensitive, did not differ reliably in SBSK. Preliminary evidence also suggests a moderating effect of sex in the relationship between SBSK and stress regulation profile membership.

### 4.1 Autonomic Profiles of Stress Responses

Converging results from the LPA and *k*‐means approach pointed to three profiles of stress regulation in young adults undergoing an attachment‐based stressor. Following the LPA findings, the largest profile (Profile 2, 79%) showed elevated parasympathetic reactivity (HRV) with moderate sympathetic activation (SCL), consistent with efficient stress regulation. This profile aligns with the depiction of the buffered profile postulated by the ACM (Del Giudice et al. 2011) and later empirically documented by Ellis et al. (2017), who also found it to be the most prevalent group in a sample of adolescents (74%). The second largest profile (Profile 1, 12%) in our study exhibited low‐to‐medium sympathetic arousal (SCL) to the stressor and the lowest parasympathetic regulatory activity (HRV), presenting a rather blunted response (or unemotional, according to the ACM). Third, the smallest emerging profile (9%) exhibited the highest sympathetic arousal (SCL) to the stressor and displayed moderate‐to‐high parasympathetic regulatory activity (HRV), matching the sensitive profile.

Our three‐profile findings align with those of Boyce and Ellis (Boyce and Ellis 2005), who also identified three reactivity groups, conceptually corresponding to sensitive, buffered, and unemotional profiles, in a sample of preschoolers using cluster analysis of cardiovascular, cortisol, and behavioral responses. In contrast, Girod et al. (2024) reported sensitive, buffered, and vigilant profiles in infants, highlighting how caregiving sensitivity predicts early autonomic calibration. While Ellis et al. (2017) detected a fourth, vigilant profile in adolescents, their latent profile indices also supported a three‐class solution. In light of this ambiguity, the authors retained the four‐profile model in accordance with the ACM's theoretical expectations.

The convergence across studies suggests that while the four‐profile taxonomy of the ACM tends to emerge under conditions of higher adversity or broader ecological range, normative populations across development often exhibit a three‐profile structure reflecting adaptive variation in autonomic regulation. It is also noteworthy that throughout the literature, stress profiles have been derived using different biobehavioral indices, ranging from hormonal (Peckins et al. 2015; Schwartz et al. 2023) and cardiovascular to electrodermal (Del Giudice et al. 2012; Ellis et al. 2017) and behavioral measures (Girod et al. 2024), and through diverse analytic approaches (e.g., cluster analysis, LPA, and hybrid methods).

Across developmental periods, the sensitive and buffered profiles have emerged as the most reliably and consistently identified profiles of autonomic responsivity, likely representing stable adaptations to supportive and moderately demanding environments. In contrast, the more dysregulated profiles, vigilant and unemotional, show greater empirical uncertainty, with their identification varying across samples and analytic models. This variability may reflect differences in developmental timing, the severity and chronicity of stress exposure, and the restricted range of adversity in most normative samples, which limit statistical power to capture the full spectrum of stress responsivity. Future research employing longitudinal and multi‐method designs in larger, more ecologically diverse cohorts will be critical to delineate how these regulatory phenotypes emerge, consolidate, and differentiate across development.

### 4.2 SBSK and Autonomic Profiles of Stress Response

In line with our hypothesis, variations in SBSK were associated with autonomic responsivity profiles across the two clustering approaches. On the 7‐point SBSK scale, all groups scored in the moderate range (3–4), reflecting the generally normative attachment representations expected in a low‐risk young‐adult sample. Individuals with higher SBSK scores (scores ≥ ∼3.8–4.0 [≈ +0.5 SD], reflecting more coherent, responsive, and elaborated secure base narratives) were more likely to exhibit a regulated autonomic profile, whereas those with lower SBSK scores, reflecting less integrated secure base organization and more limited expectations of caregiver availability, were more likely to fall into the unemotional profile. Specifically, each one‐point increase in SBSK was associated with roughly halved odds of belonging to the unemotional profile relative to the buffered profile, about a 51% reduction in the latent profile solution (OR = 0.49) and about a 55% reduction in the *k*‐means solution (OR = 0.45). The buffered and sensitive profiles, particularly, are best understood as adjacent regions on a continuum of sympathetic arousal rather than as discrete categories. *K*‐means, which favors clusters of comparable size, set the dividing line lower on this gradient and reclassified 34 moderate‐arousal buffered cases as sensitive, while the high‐arousal core of the sensitive profile remained stable across methods. The participants who shifted from buffered to the *K*‐means sensitive cluster were distinguished mainly by higher sympathetic arousal, while maintaining comparable parasympathetic regulation. Moreover, SBSK did not change, neither distinguishing the reassigned participants from those who stayed buffered or from the sensitive group, nor differing between the buffered and sensitive groups overall. These findings suggest that individuals with more elaborated secure‐base scripts are more likely to show balanced arousal regulation under attachment‐relevant stress. This aligns with attachment theory's expectation that internalized secure‐base expectancies scaffold efficient mobilization and recovery when stress is mild to moderate (Mikulincer and Florian 2003).

Our results align with recent findings by Qu and Leerkes (2018), who reported that attachment avoidance predicts reduced sympathetic and parasympathetic activity during attachment‐related stress, consistent with the unemotional profile. Similarly, Girod et al. (2024) found that higher maternal sensitivity, a hallmark of attachment security, predicted the emergence of buffered and sensitive autonomic profiles in infancy, illustrating the developmental continuity of attachment calibration from early caregiving to adulthood. Taken together, these findings suggest that secure attachment representations foster adaptive physiological regulation during social stress, whereas less secure or avoidant representations reflect dysregulated profiles.

Treating these profiles as the lasting trace of early calibration assumes that autonomic individual differences are stable enough to carry an early imprint forward, which this study cannot test. Building on earlier evidence, the stability that has been shown is mainly in resting tone (Calkins and Keane 2004; El‐Sheikh 2005; Hinnant et al. 2011; Seidman et al. 2024), while the profiles here are based on reactivity and regulation, which are less stable over development (Bornstein and Suess 2000; Alkon et al. 2011; Dollar et al. 2020), and the electrodermal measure that sets the sensitive profile apart has the least developmental evidence of all (Crider 2008). Because we measured attachment and physiology simultaneously in adulthood, we treat these profiles as adult individual differences whose developmental origins are plausible but not established here, and longitudinal studies that follow autonomic function from childhood are warranted to test continuity directly.

A key contribution of the present study lies in examining attachment‐related regulation through *multiple autonomic indices*, rather than relying on bivariate associations. Human stress regulation reflects the coordinated interplay between the parasympathetic and sympathetic systems, yet most prior work has relied on bivariate correlations that capture only fragments of this dynamic process. In our data, simple correlations revealed a modest link between SBSK and parasympathetic reactivity (*r* = 0.19), but no relation to sympathetic activity. Similar patterns have been reported in recent studies using the ASA. Both Haydon and Groh (2024) and Groh et al. (2024) found that higher SBSK scores were associated with greater parasympathetic activation but unrelated to sympathetic responding.

While these variable‐centered findings converge with ours, they also highlight the limitations of single index approaches, which identify direction but not configuration. By modeling the activity of both autonomic branches, our person‐centered analyses revealed distinct regulatory profiles that integrate activation and regulation within a comprehensive system. This multidimensional approach, borrowed from the ACM, captures the complexity of human stress responsivity more faithfully and shows how individual differences in attachment shape autonomic balance.

### 4.3 Sex Differences

Exploratory interaction analyses suggested that the association between SBSK and autonomic profile membership may vary by sex, emerging in the LPA but not the *k*‐means solution. However, the sex moderation observed for the LPA profiles should be read with caution given the small subgroup sizes and its absence in the *k*‐means solution. We therefore regard it as hypothesis‐generating, to be further corroborated. Within the sensitive profile, characterized by concurrent high parasympathetic (HRV) and high sympathetic (SCL) activation, males exhibited lower SBSK than females, whereas no sex differences were observed in the buffered or unemotional profiles. As illustrated in Figure 4, this pattern should be regarded as descriptive and tentative, given the small subgroup sizes and exploratory design. The distribution suggests that among individuals showing higher regulatory capacities, females provided more elaborated secure base representations. In contrast, males tended to exhibit comparable physiological activation, albeit with less elaborated attachment scripts. This aligns with theories of sex‐differentiated stress calibration (Del Giudice et al. 2011; Ellis et al. 2017), proposing that males and females develop distinct autonomic configurations under comparable developmental conditions, influenced by hormonal sensitivity and socialization processes. Females’ stress systems are thought to favor social engagement and co‐regulation (Taylor et al. 2000), whereas males’ responses emphasize autonomic mobilization and vigilance (for a review, see Kudielka and Kirschbaum 2005). Sociocultural display rules that discourage emotional expressiveness in men (Chaplin et al. 2017) may further reinforce these patterns.

### 4.4 Strengths and Limitations

This study offers several key strengths. First, it employed an ethnically diverse sample, thereby enhancing the generalizability of the findings across cultural contexts that are often underrepresented in psychophysiological research. Second, the study leveraged complementary statistical approaches, LPA and *k*‐means clustering, to derive convergent, person‐centered classifications of autonomic responsivity, strengthening confidence that the identified profiles reflect a robust, underlying organization rather than model‐specific artifacts. Third, the study incorporated multiple physiological indices of stress reactivity, HRV and SCL, capturing both parasympathetic and sympathetic processes to provide a more integrated picture of autonomic coordination during an attachment‐relevant stress. One limitation concerns statistical power. The a priori calculation used a multiple regression benchmark rather than the latent profile and multinomial models that supported our primary inferences, and a Monte Carlo sensitivity analysis indicated that the design was adequately powered to recover the dominant profile structure and to detect only large associations with profile membership. More modest effects and reliable inference within the smallest profiles fell outside its sensitivity. The significant association we report should be read as a large but imprecisely estimated effect that awaits replication in larger samples. This is particularly true for the exploratory sex differences, wherein the sensitive profile rests on a small number of males and females, and we therefore present it as a hypothesis‐generating observation that requires validation in larger and more balanced samples.

Future research should aim to replicate these findings and examine the stability of autonomic stress profiles across different stress contexts, such as agonistic versus non‐agonistic or social‐evaluative tasks, which may recruit distinct regulatory strategies. Testing whether similar configurations emerge across these conditions will clarify whether the observed profiles represent generalized or context‐specific regulatory imprints of early attachment experiences. Longitudinal research is also necessary to investigate the temporal stability of these profiles and to examine how early caregiving and attachment representations predict autonomic calibration across development and into adulthood.

In addition, future studies should adopt a multisystem perspective, integrating autonomic measures with hypothalamic–pituitary–adrenal (HPA) axis activity to capture the coordination between peripheral and endocrine stress systems. Assessing sympathetic and parasympathetic activity within the same organ system, for example, cardiac pre‐ejection period (PEP) alongside HRV, could reveal subtle timing dynamics in reciprocal control (Kaeppler and Erath 2025). Further, incorporating recovery trajectories alongside responsivity will enable a more comprehensive assessment of adaptive regulation and resilience mechanisms.

### 4.5 Conclusion

This study provides the first evidence that distinct autonomic stress profiles are identifiable in adulthood and are linked to attachment security. By integrating attachment theory with the adaptive calibration model, the findings suggest that the regulatory imprint of attachment relationship quality shapes autonomic coordination later in life, with implications for early child development and mental health across the lifespan. These findings underscore the importance of a sensitive caregiving ecology, beyond instrumental care, in shaping stress regulation strategies. Accordingly, this line of work can inform early childhood programs, highlighting the value of awareness and interventions for parents to foster emotional attunement, responsiveness, and co‐regulation, not only to promote secure attachment but also to support healthy physiological self‐regulation. Educators and early care professionals may similarly benefit from training that emphasizes emotion‐regulatory support within classroom settings. At the level of mental health, the results suggest that attachment‐based and emotion‐focused therapies targeting regulatory flexibility may help recalibrate dysregulated autonomic patterns rooted in early relational experiences. Together, our findings suggest that caregiving quality is both a psychosocial and a biological scaffold for stress adaptation.

## Author Contributions

**Stefania V. Vacaru**: conceptualization, data curation, analysis and interpretation, supervision, writing – original draft. **Victoria L. Zhu**: conceptualization, data collection, writing – review and editing. **Lukas Spiess**: data curation and visualizations, writing – review and editing. **Aicha Mazrina**: data analysis and visualizations, writing – review and editing. **Greeshma Giridas**: data collection and curation, writing – review and editing. **Theodore E. A. Waters**: conceptualization, resources, supervision, writing –review and editing.

## Funding

This study was funded by the NYU Research Catalyst Grant, awarded to Theodore E. A. Waters.

## Ethics Statement

The study was approved by the NYUAD Institutional Review Board (HRPP‐2023‐46) and was performed in accordance with the World Medical Association Declaration of Helsinki.

## Consent

Participants provided written informed consent, and the privacy rights of human subjects were observed.

## Conflicts of Interest

The authors declare no conflicts of interest.

## Data Availability

Data will be made available on request. The preprocessing code for the physiological data supporting this study's findings is available on GitHub.
